# Effectiveness of high-risk human papillomavirus genotyping for cervical cancer screening. A multicentre screening cohort study in rural China

**DOI:** 10.23938/ASSN.1065

**Published:** 2024-06-11

**Authors:** Yan-Qin Yu, Ming-Yue Jiang, Xun Zhang, Qin-Jing Pan, Le Dang, Rui-Mei Feng, Nasra Mohamoud Ali, Wen Chen, You-Lin Qiao

**Affiliations:** 1 Baotou Medical College Clinical Epidemiology Research Centre of The First Affiliated Hospital of Baotou Medical College Department of Public Health and Preventive Medicine. Baotou China; 2 National Cancer Centre/National Clinical Research Centre for Cancer/Cancer Hospital. Chinese Academy of Medical Sciences and Peking Union Medical College Department of Cancer Epidemiology Beijing China; 3 First Affiliated Hospital of Dalian Medical University Department of Oncology Dalian China; 4 School of Population Medicine and Public Health Chinese Academy of Medical Sciences and Peking Union Medical College Beijing China

**Keywords:** Papillomavirus Infections, Uterine Cervical Neoplasms, Mass Screening, Rural Areas, China, Infección por Virus del Papiloma Humano, Cáncer de cuello uterino, Cribado, Zonas Rurales, China

## Abstract

**Background:**

This study aimed to assess the effectiveness of high-risk human papillomavirus (HR-HPV) primary testing for cervical cancer screening in China’s rural areas.

**Methods:**

Women aged 21-64 years were recruited. Cervical cytology was diagnosed following the Bethesda 2001 classification system, HPV infection (HR-HPV, HPV-16, HPV-18, and other 12 genotypes) identified by Cobas-4800, and colposcopy and biopsy performed when required. Primary outcomes were defined as the cumulative incidence of cervical intraepithelial neoplasia grade 2/3/higher (CIN2/3+) and its relative risk at baseline and at the 36-month follow-up.

**Results:**

The study included 9,218 women; mean age was 45.15 years (SD: 8.74); 81% completed the follow-up. The most frequent type of cytological lesions (12.4% ) were ASCUS (8.4%) and LSIL (2.2%). HR-HPV infection (16.3%) was more prevalent in HPV-16 than in HPV-18 (3 vs 1.5%); a positive relationship with the severity of the lesions, from 29.8% in ASCUS to 89.6% in HSIL was found. At baseline, 3.5% of the patients underwent colposcopy; 20% had a positive diagnosis. At the 36-month follow-up, the cumulative incidences of CIN2+ and CIN3+ were higher in women with HR-HPV infection (16.9 vs 0.5% and 8.2 vs 0.2%). The relative risk of CIN2/3+ was lower in HR-HPV-negative women compared to those with a negative cytology at baseline (0.4; 95%CI: 0.3-0.4).

**Conclusions:**

High-risk HPV-based screening may significantly reduce the risk of CIN2/3+ compared with cytology testing. This may be a new resource for public health demands in China’s rural areas.

## RESUMEN

**Fundamento:**

El objetivo es evaluar la viabilidad del cribado de cáncer de cérvix mediante determinación del virus del papiloma humano (VPH) de alto riesgo (AR) en China rural.

**Métodos:**

Se reclutaron mujeres de 21 a 64 años. La citología cervical se informó siguiendo la nomenclatura Bethesda 2001. La infección por VPH (VPH-AR, VPH-16, VPH-18 y otros 12 genotipos) se identificó mediante Cobas-4800. Algunos resultados exigieron la realización de colposcopia y biopsia. Los resultados primarios fueron la incidencia acumulada de neoplasia intraepitelial cervical de grado 2/3/superior (CIN2/3+) y su riesgo relativo (RR) al inicio y a los 36 meses de seguimiento.

**Resultados:**

El estudio incluyó 9.218 mujeres; la edad media fue 45,15 años (DE: 8,74) y el 81% completó el seguimiento. Las lesiones citológicas (12,4%) más frecuentes fueron ASCUS (8,4%) y LSIL (2,2%). La infección por VPH-AR (16,3%) fue más prevalente por VPH-16 que por VPH-18 (3 vs 1,5%) y aumentó con la gravedad de las lesiones (ASCUS 29,8% vs HSIL 89,6%). Al inicio se realizaron 3,5% colposcopias, el 20% patológicas. A los 36 meses de seguimiento, la incidencia acumulada de CIN2+ y CIN3+ fue mayor en mujeres con VPH-AR positivo (16,9 vs 0,5% y 8,2 vs 0,2%). El RR de CIN2/3+ fue menor en mujeres con VPH-AR negativo que con citología negativa al inicio del estudio (0,4; IC95%: 0,3-0,4).

**Conclusiones:**

El cribado de cáncer de cérvix mediante VPH-AR parece reducir significativamente el riesgo de CIN2/3+ respecto a la citología, por lo que podría ser un nuevo recurso de salud pública en China rural.

## INTRODUCTION

Cervical cancer is a serious public health problem for women worldwide, with around 604,000 new cases and 342,000 deaths globally in 2020^1^. In China, 119,300 new cases and 37,200 deaths were registered in 2022^2^. Therefore, cervical cancer prevention and control is a major public health challenge in China.

Cervical cancer screening to detect and treat precancerous lesions before they progress to invasive cancer^3^^,^^4^ is an effective strategy to reduce its incidence and mortality when screening coverage is over 70% of the target population^5^. Due to the extensive development of cervical cancer screening, the incidence of cervical cancer in the USA has dropped by over 50% in the past 30 years^1^. However, its incidence in developing countries has not been effectively reduced due to the lack of standardisation and insufficient coverage^6^. Although close to 80 million women have been screened in China’s rural areas in the past decade, this represents less than 30% of the female population in this country^7^, far from the screening target of 70% proposed by the WHO. 

Conventional cervical cancer screening methods include cytology testing (Pap smear), visual ins-pection with acetic acid (VIA), or Lugol’s iodine (VILI)^8^, among others. Rural areas in China have a large population base, shortage of health resour-ces and cytopathologists, and low economic base and financial strength. In some areas, examinations by cytopathologists have been abandoned, and VIA/VILI have been reintroduced as the primary screening methods. Cervical cancer naked eye visual screening can solve the above problems allowing to identify some cases, but with a low detection rate^9^. This has prevented the achievement of high-quality cervical cancer screening^6^. Therefore, there is an urgent need for new screening methods to ensure a high-quality cervical screening in women aged 20-64 years and extend the time interval for cervical cancer screening.

One of the promising screening methods is human papillomavirus (HPV) genotyping, which can identify the specific types of HPV associated with a higher risk of cervical cancer, such as genotypes 16 (HPV 16) and 18 (HPV 18)^9^. HPV genotyping has several advantages: higher sensitivity, longer screening interval, and better triage of women with abnormal cytology or HPV-positive results, compared with other screening methods^10^^-^^13^; thus, HPV testing has replaced cytology as the leading cervical cancer screening technique worldwide^14^^-^^16^.

A multicentre open-label randomised clinical trial^17^ reported that high-risk human papillomavirus (hrHPV) testing in a national program in China was superior to cytology and VIA/VILI as the initial test in routine screening in primary care centres. However, although the national programme has facilitated the development of primary health centres by improving infrastructures and increasing the personnel, the number of qualified cytologists and gynaecologists is still low in rural China^18^ and HPV genotyping is not widely used for cervical cancer screening in this country. Moreover, there is a lack of data on the performance and feasibility of HPV genotyping as a primary screening method in rural China, where the epidemiology and risk factors of cervical cancer may differ from urban areas. 

Positive rates in HPV screening with a colposcopy biopsy for CIN2+ detection are higher than with Pap smears, thin-layer cytology, or DNA ploidy detection, reducing the omission of cervical lesions, providing a reference basis for the early clinical diagnosis and treatment, and minimising unne-cessary colposcopy procedures^19^. Although the po-sitive predictive value is low (23.96%) and the cost of short-term screening high, the cost of long-term screening is the lowest; therefore, it is more practical in some less economically developed areas^20^^,^^21^. Therefore, new cervical cancer screening strategies and methods are needed for these areas. 

This study aimed to evaluate the utility of HPV genotyping as the primary screening method for cervical cancer in rural China and compare the outcomes with cytological results. The primary outcomes of this study were to assess the cumulative incidence and relative risk of cervical intra-epithelial neoplasia grade 2 or higher and grade 3 or higher (CIN2/3+) among women with different HPV genotypes and cytological results at baseline and at 36 months of follow-up.

## SUBJECTS AND METHODS

Multicentre, open-label, observational cohort study. Three rural areas - Ordos in Inner Mongolia (Hangjin Banner and Yi Jinholo Banner), the Shanxi Province (Xiangyuan County), and the Zhejiang Province (Jinyun County and Jingning County) - were selected as the study areas. The study was conducted between January 2016 and January 2019. 

Inclusion criteria: female participants 1) aged 21-64 years, 2) without a history of cervical cancer, hysterectomy, or pelvic radiotherapy (e.g., for the treatment of cervical cancer), and 3) who were not pregnant during the study period. Exclusion criteria: women 1) with severe autoimmune diseases or uraemia, 2) who had received or planned to receive HPV vaccination, or 3) who did not complete the questionnaires on demographic and clinical information or refused gynaecological examinations. 

The rural areas were selected based on the following criteria: 1) high incidence of cervical cancer according to China´s National Cancer Registry data, 2) low coverage of cervical cancer screening according to the statistics of China´s National Health Commission, and 3) representative distribution of ethnic minority groups, such as Mongolian. The aim of selecting these rural areas was to evaluate the utility of HPV genotyping in a high-risk, low-resource diverse population.

All the participants signed a written informed consent before being included in the study. They agreed to voluntarily undergo the required gynaecological examinations and to the publication of the results. This study was approved by the Cancer Hospital, Chinese Academy of Medical Sciences (Ref. #: 16-013/1092) and by all the institutional review boards of the participating hospitals.

Trained local physicians performed the necessary procedures. The authors asked for the collaboration of doctors of the local hospitals from the study areas to perform the screenings and assessment by a superior department was allowed. 

The interviews to gather demographic information and perform a gynaecological examination were made face-to-face and individually. 

The following demographic data were collected: age (years), categorized by 5-year age groups from 21 to 65; marital status: unmarried, married, widowed, separated, divorced, other; education: none, primary school, junior high school, high school, university and above. All patient information was de-identified and known only to the authors and the screening physicians. 

*Cervical cytology.* Exfoliated cervical cells were obtained using ThinPrep^®^ medium (Hologic^TM^ Inc., San Diego, USA). The cytological results were reported using the 2001 Bethesda system^22^^,^^23^: ASCUS: atypical squamous cells of undetermined significance, ASC-H: high-grade squamous intraepithelial lesion, LSIL: low-grade squamous intraepithelial lesion, HSIL: high-grade squamous intraepithelial lesion, or AGCs: atypical glandular cells. If the results of the cytology were ASCUS or higher, colposcopy and biopsy of the suspicious lesions were ordered. Participants who tested negative for intraepithelial lesions or malignancy (NILM) were deemed to have screened negative. Unsatisfactory smears were those for which the diagnosis of epithelial abnormalities was uncertain. 

*Human papillomavirus screening strategy.* The exfoliated cervical cells obtained using ThinPrep^®^ medium were also tested for hrHPV using a polymerase chain-reaction-based Cobas 4800 test (Roche Diagnostics GmbH, Mannheim, Germany). The Cobas results were negative/positive for HPV 16 individually, HPV 18 individually, and for a pool of other 12 HPV genotypes (31, 33, 35, 39, 45, 51, 52, 56, 58, 59, 66, and 68)^24^^,^^25^. Women with one or more HPV screening results positive for HPV 16 or HPV 18 were classified as HPV 16/18 positive.

**Figure 1:**
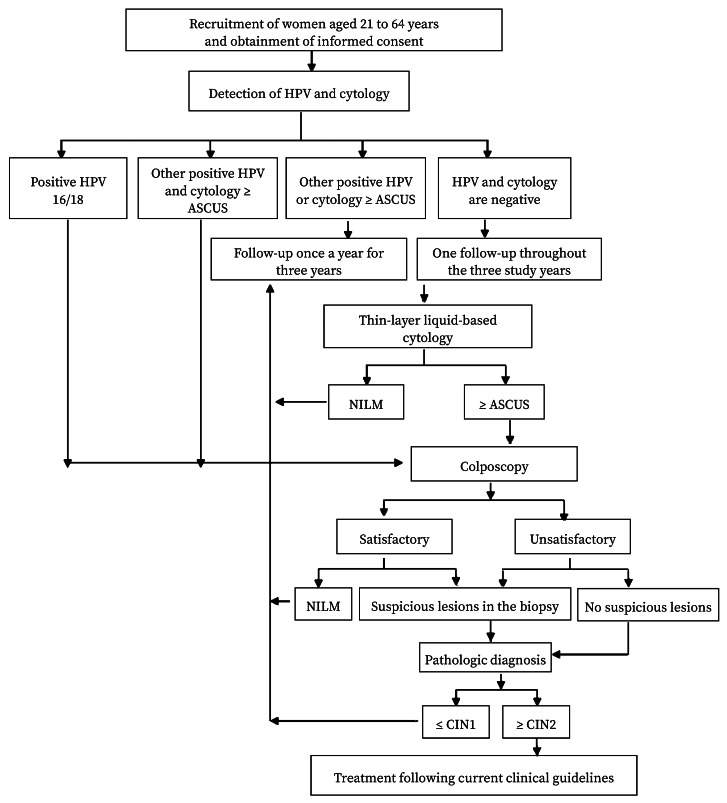
Flowchart of cervical cancer screening among women aged 21 to 64 years.

HPV: human papillomavirus; ASCUS: atypical squamous cells of undetermined significance; TCT: thin-layer liquid-based cytology; NILM: intraepithelial lesions or malignancy; ≤ CIN1: mild dysplasia or normal result; ≥ CIN2: moderate or severe dysplasia or cervical cancer.

HPV 16/18-positive or HPV positive women with a cytological diagnostic of ASCUS and above were directly examined by colposcopy and a biopsy was taken from women with an abnormal colposcopy. Women diagnosed at least of atypical squamous cells (ASYCs, a cytological category that includes ASCUS and atypical squamous cells that cannot exclude an ASC-H) or who were HPV-positive underwent further tests (Fig. 1).

*Colposcopy and biopsy* sampling were performed when required, as described earlier^26^. To carry out the procedure, the physician had to make sure the patient was in the lithotomy position fully exposing the cervicovaginal area and then wipe the cervical secretion with a sterile cotton ball, adjust the eyepiece refraction of the colposcope, and focus the colposcope to the optimal state. Furthermore, the physician had to fully expose the transformation zone, epithelium and blood vessels, and capture an original image of the cervix. Next, 3% acetic acid cotton balls were rubbed on the cervical surface for approximately 30 seconds and images captured at 1 and 3 minutes. A compound iodine solution was then evenly applied to the cervical surface and addi-tional images taken at 1 and 3 minutes. Cervical tissue was taken from negative iodine test areas or suspicious lesion areas, fixed with 10% formaldehyde solution, and promptly sent for pathological examination. If colposcopy revealed no significant abnormalities, a four-quadrant biopsy with cervical curettage was performed and sent for pathological examination.

*Pathological examination*. Biopsies were assessed by two senior pathologists and a diagnosis made following the CIN nomenclature system as follows: NILM (negative for intraepithelial lesion malignancy), CIN1 (mild dysplasia), CIN2 (moderate dysplasia), CIN3 (severe dysplasia), and cervical cancer plus vulvar intraepithelial neoplasia. Patients with lesions diagnosed with at least CIN2 (CIN2+) or at least CIN3 (CIN3+) each year, positive primary screening results for HPV, and positive cytology, were also scheduled for intervention-free follow-up visits. During follow-up visits, colposcopies were performed on all participants who had a positive cytology in the same manner as the initial baseline cervical cancer screening.

*Statistical analysis.* A database was created using Microsoft Access 2007. Statistical analyses were performed with SPSS version 25.0. Qualitative and quantitative variables are presented as frequency / percentage (%) and mean / standard deviation (SD), respectively. The Chi-squared (χ^2^) test was used to compare the characteristics of participants and the cumulative incidence rates of hrHPV, HPV 16, HPV 18, HPV 16/18, and the other 12 high-risk types and cytology. The risk ratio (RR) of hrHPV, HPV 16, HPV 18, HPV 16/18, and the other 12 high-risk types and cytology were calculated together with 95% confidence intervals (CI) using the Newcombe-Wilson method. All tests were two-tailed and the level of significance was set at p < 0.05 or CI of the ratios below or above 1.

## RESULTS

Nine thousand five hundred women aged 21-64 years living in villages or sub-districts were invited to participate in the study; all signed the written informed consent. After applying the selection criteria, 282 women were excluded; 9,218 eligible women were interviewed and underwent gynaecological examinations / laboratory tests. Participation and eligibility rates were 100% and 97%, respectively.

**Table 1:** Demographic characteristics of the women enrolled in the study (n=9,218)

Variable	n	%
Age group (years)		
21-25	45	0.5
26-30	482	5.2
31-35	922	10.0
36-40	1,327	14.4
41-45	1,845	20.0
46-50	1,86	20.2
51-55	1,57	17.0
56-60	862	9.4
61-65	305	3.3
Marital status		
Unmarried	10	0.1
Married	9,034	98.0
Widowed	126	1.4
Separated	12	0.1
Divorced	32	0.4
Others	4	0.04
Education level		
None	641	7.0
Primary school	1,885	20.4
Junior high school	3,707	40.2
High school	1,398	15.2
University or above	1,587	17.2

Mean age was 45.15 years (SD: 8.74), the age group 41 to 55 years included the larger number of women (n = 1,860; 57.2%). Almost all women were married (98%) and 40.2% of all participants had undergone formal education until junior high school (Table 1). 

### Distribution of cytology results based on human papillomavirus status at baseline

An abnormal cytology was found in 1,152 women (12.4%) at baseline, ASCUS (8.4%) and LSIL (2.2%) being the most frequently diagnosed lesions. Eight cases of cervical cancer (0.1%) were identified. Unsatisfied smears were obtained in less than 1% of the cases (Table 2).

Overall, prevalence of hrHPV infection was 16.3%, prevalence of HPV 16 doubled that of HPV 18 (3 vs 1.5%, respectively), and prevalence of the other 12 high-risk genotypes was 16.3%. Coinfection by different HPV genotypes was observed. Prevalence of hrHPV positively associated to the severity of cytology lesions was 29.8% in ASCUS and 89.6% in HSIL. In women with squamous cell carcinoma, hrHPV infection was found in 34.8% of the cases. Infection with HPV 16, HPV 18, or HPV 16/18 was associated with higher severity of cytological lesions (χ^2^ = 0.738, *p* = 0.037), whereas no changes were seen for the infection rates of the other 12 hrHPV genotypes. Prevalence of hrHPV in NILM was 12.1%, while in women with ≥ ASCUS it was 55.25% (n = 625). hrHPV-negative women comprised 0.6% of all LSIL participants versus 55.3% in ≥ LSIL (Table 2). 

**Table 2:** Frequency of cytology results based on HPV status at baseline

Cytology	Positive HPV					Negative HPV (n%)	Global (n%)
	HR (n%)	16 (n%)	18 (n%)	16/18 (n%)	Other HR (n%)		
NILM	969 (12.1)	152 (1.9)	90 (1.1)	234 (2.9)	812 (10.1)	7,037 (87.9)	8,006 (86.9)
ASCUS	230 (29.8)	40 (5.2)	17 (2.2)	56 (7.3)	197 (25.6)	541 (70.2)	771 (8.4),
LSIL	153 (73.9)	25 (12.1)	11 (5.3)	35 (16.9)	133 (64.3)	54 (26.1)	207 (2.2)
ASC-H	57 (86.4)	22 (33.3)	5 (7.6)	25 (37.9)	43 (65.2)	9 (13.6)	66 (0.7)
HSIL	69 (89.6)	31 (40.3)	8 (10.4)	37 (48.1)	50 (64.9)	8 (10.4)	77 (0.8)
SCC	8 (34.8)	5 (21.7)	1 (4.3)	6 (26.1)	4 (17.4)	0	23 (0.2)
AGC	10 (43.5)	1 (4.3)	3 (13.1)	4 (17.4)	8 (34.8)	13 (56.5)	23 (0.2)
Unsatisfactory	8 (13.3)	2 (33.3)	1 (16.7)	3 (5.0)	6 (10.0)	52 (86.7)	60 (0.7)
Global	1504 (16.3)	278 (3.0)	136 (1.5)	400 (4.3)	1,253 (13.6)	7,714 (83.7)	9,218

HPV: human papillomavirus; HR: high-risk genotypes; HSIL+: high-grade squamous intraepithelial lesion or worse; LSIL: low-grade squamous intraepithelial lesion; ASCUS: atypical squamous cells of undetermined significance; NILM: negative for intraepithelial lesions or malignancy; ASC-H: high-grade squamous intraepithelial lesion; AGC: atypical glandular cell; SCC: squamous cell carcinoma; Other: 31, 33, 35, 39, 45, 51, 52, 56, 58, 59, 66, and 68 high-risk HPV genotypes.

### Cumulative incidence rate of cervical intraepithelial neoplasia grade 2/3+ based on hrHPV screening at baseline

Considering the cytology results at baseline, 1,249 women had to undergo a colposcopy (13.5%). Two hundred forty-eight women (19.9%) obtained a pathological diagnosis post-colposcopy at baseline: 108 (8.6%) cases of CIN1, 66 (5.3%) cases of CIN2, 68 (5.4%) cases of CIN3, three cases (0.24%) of cervical cancer, and three cases (0.24%) of vulvar intraepithelial neoplasia (0.24%) were identified. 

Almost half of the cases were hrHPV-positive (n = 527; 42.2%), mostly HPV 16 (n = 124; 9.9%) and the other 12 hrHPV genotypes (n = 435; 34.8%). As shown in Table 3, all CIN2+ and CIN3+ lesions were positive for hrHPV, with a trend similar to that of hrHPV. 

Women with HPV infection showed higher risk of CIN2+ when infected with hrHPV, HPV 16, HPV 18, HPV 16/18, or the other 12 hrHPV types; the same results were seen for the RR of CIN3+. On the other hand, women with an abnormal cyto-logy showed lower risk of CIN2+ and CIN3+ when infected with HPV 16,and higher RR of CIN2+ for the other hrHPV group (Table 3).

**Table 3:** Cumulative incidence rate of colposcopy outcomes at baseline based on hrHPV testing and cytology

HPV	CP n (%)	CIN2+			CIN3+		
		n (%)	RR (95%CI)		n (%)	RR (95%CI)	
			HPV testing	Cytology		HPV testing	Cytology
HR	527	66	9.3	1.2	68	4.8	1.1
	(5.7)	-100	(7.8-11.1)	(1.0-1.4)	-100	(4.0-5.7)	(0.9-1.3)
16	124	39	64.1	0.8	43	68.0	0.8
	(1.3)	(59.1)	(53.2-76.5)	(0.7-0.9)	(63.2)	(57.1-80.2)	(0.7-0.9)
18	45	5	6.1	0.8	4	4.0	1.1
	(0.5)	(7.6)	(2.5-14.6)	(0.3-2.1)	(5.9)	(1.2-13.3)	(0.3-4.0)
16/18	163	43	52.7	0.8	47	57.1	0.8
	(1.8)	(65.2)	(42.1-65.9)	(0.6-1.0)	(69.4)	(46.0-70.8)	(0.6-1.0)
Other	435	42	11.9	1.5	38	8.2	1.3
	(4.7)	(63.6)	(9.5-14.9)	(1.2-1.8)	(55.9)	(6.5-10.4)	(1.0-1.7)

CIN2+: at least moderate dysplasia; CIN3: at least severe dysplasia; CP: colposcopy; RR: relative risk; CI: confidence interval; HPV: human papillomavirus infection; HPV testing: human papillomavirus positive *vs* negative; Cytology: abnormal *vs* normal; HR: high risk HPV; 16/18: positive for HPV-16 and/or HPV-18; Other: 31, 33, 35, 39, 45, 51, 52, 56, 58, 59, 66, 68 hrHPV genotypes.

### Cumulative incidence rate of cervical intraepithelial neoplasia grade 2/3+ based on hrHPV screening at Month 36 of the follow-up

After 36 months, 7,516 women (81.5%) had completed the follow-up treatment post-cervical cancer screening. 

Women with HPV infection at Month 36 showed higher incidence rate of CIN2+ when infec-ted with any genotype of hrHPV (16, 18, 16/18, or other); lower but similar results were observed for CIR of CIN3+. The risk for CIN2+ in HPV 16- and HPV 18-positive women at Month 36 were 8.5 and 3.3 times higher, respectively, than those of the HPV-negative group. Moreover, women with negative baseline HPV tests showed lower risk of CIN2+ and CIN3+ (Table 4).

**Table 4:** Cumulative incidence rate of colposcopy outcomes at 36-month follow-up based on hrHPV testing and cytology

HPV	CP n (%)	CIN2+			CIN3+		
		n (%)	RR (95%CI)		n (%)	RR (95%CI)	
			HPV testing	Cytology		HPV testing	Cytology
HR	782	132	33.8	0.3	64	38.9	0.2
	(10.4)	(16.9)	(27.2-42.1)	(0.2-0.5)	(8.2)	(30.5-49.6)	(0.1-0.4)
16	586 (7.8)	251	85.4	0.3	140	113.8	0.4
		(42.9)	(72.3-100.8)	(0.2-0.5)	(23.9)	(95.1-135.9)	(0.2-0.7)
18	849	148	34.7	0.05	52	28.9	0.01
	(11.3)	(17.4)	(25.8-46.5)	(0.01-0.2)	(6.1)	(20.2-41.5)	(0.001-0.05)
16/18	661	230	69.4	0.4	123	88.3	0.5
	(8.8)	(34.8)	(55.9-86.2)	(0.3-0.6)	(18.6)	(70.1-111.7)	(0.3-0.8)
Other	834	118	28.4	0.7	52	29.6	0.5
	(11.1)	(14.2)	(22.5-35.9)	(0.5-1.0)	(6.2)	(22.0-39.8)	(0.3-0.8)
Negative	436	2	-	0.4	1	-	0.4
	(5.8)	(0.5)		(0.3-0.6)	(0.2)		(0.2-0.7)

CIN2+: at least moderate dysplasia; CIN3: at least severe dysplasia; CP: colposcopy; RR: relative risk; CI: confidence interval; HPV: human papillomavirus infection; HPV testing: human papillomavirus positive vs negative; Cytology: abnormal *vs* normal; HR: high risk HPV; 16/18: positive for HPV-16 and/or HPV-18; Other: 31, 33, 35, 39, 45, 51, 52, 56, 58, 59, 66, 68 hrHPV genotypes.

As for the cytological results, women with an abnormal cytology showed lower risk of CIN2+ when infected with hrHPV, HPV 16, HPV 18, or HPV 16/18.

### Cumulative incidence rate of cervical intraepithelial neoplasia grade 2/3+ with cytology and human papillomavirus screening at Month 36 of the follow-up

After 36 months of follow-up, cumulative incidences of CIN2/3+ increased alongside the malignancy grade diagnosed in the cervical cytology (Table 5).

Combining the cytology and HPV screening data, the cumulative incidence rate of CIN2/3+ was higher in women with a positive cytology for hrHPV in comparison to HPV-negative ones; e.g., the cumulative incidence of CIN3+ in HSIL cytology increased from 28.6% if HPV was negative to 56.1% in hrHPV-positive women. The highest cumulative incidence rate of CIN2/3+ was observed for HPV 16 (Table 5).

**Table 5:** Cumulative incidence rates of cervical intraepithelial neoplasia grade 2/3/higher combined with cytology and HPV screening

		NILM	ASCUS	LSIL	HSIL	AGC	ASC-H	SCC
CIN2+ cumulative incidence	HPV	1.2	6.7	15.2	80.8	20.0	48.3	75.0
	HR	77 (7.9)	38 (16.5)	30 (19.4)	57 (83.3)	4 (44.4)	29 (50)	6 (75.0)
	Negative	14 0.2)	12 (2.2)	2 (4.1)	5 (57.1)	0	3 (37.5)	0
	16	36 (23.8)	18 (45.2)	11 (45.8)	28 (90.3)	1 (100.0)	14 (63.2)	5 (100.0)
	18	10 (10.8)	4 (12.5)	2 (18.2)	8 (100)	2 (50.0)	0	0
	16/18	44 (18.9)	19 (34.8)	13 (38.2)	34 (91.7)	3 (75.0)	14 (57.1)	3 (57.1)
	Other	46 (5.7)	32 (16.0)	21 (15.5)	42 (83.3)	4 (55.6)	20 (47.5)	2 (47.5)
	Global	112 (1.2)	72 (6.7)	62 (15.2)	164 (80.8)	8 (20.0)	4 (48.3)	18 (75.0)
CIN3+ cumulative incidence	HPV	0.6	1.5	5.6	53.4	15.0	26.7	37.5
	HR	35 (3.6)	10 (4.4)	9 (6.2)	39 (56.1)	3 (33.3)	16 (28.8)	3 (37.5)
	Negative	7 (0.1)	1 (0.2)	2 (4.1)	2 (28.6)	0	1 (12.5)	0
	16	20 (13.1)	5 (12.9)	3 (12.5)	23 (74.2)	1 (100.0)	7 (31.6)	3 (60.0)
	18	1 (1.4)	1 (6.3)	1 (9.1)	3 (42.8)	2 (50.0)	0	0
	16/18	22 (9.2)	6 (10.9)	4 (11.7)	26 (69.4)	3 (75.0)	7 (28.6)	2 (28.6)
	Other	18 (2.2)	9 (4.4)	7 (5.5)	23 (45.8)	4 (44.4)	12 (27.5)	1 (27.5)
	Global	56 (0.6)	16 (1.5)	23 (5.6)	108 (53.4)	6 (15.0)	2 (26.7)	9 (37.5)

CIN2+: at least moderate dysplasia; CIN+: at least severe dysplasia; NILM: negative for intraepithelial lesions or malignancy; ASC-H: high-grade squamous intraepithelial lesion; AGC: atypical glandular cell; SCC: squamous cell carcinoma; HPV: human papillomavirus infection; hrHPV: high-risk human papillomavirus; 16/18: positive for HPV-16 and/or HPV-18; Other: 31, 33, 35, 39, 45, 51, 52, 56, 58, 59, 66, and 68 hrHPV genotypes; HSIL+: high-grade squamous intraepithelial lesion or worse; LSIL: low-grade squamous intraepithelial lesion; ASCUS: atypical squamous cells of undetermined significance

## DISCUSSION

This study presents the results of cervical cancer screening by cytology and HPV-related to CIN2/3+ detection at baseline and 36 months after the initial screening. To the best of our knowledge, this is the first cohort study with a large population and a multicentre design in which HPV genotype testing is used for cervical cancer screening in minority populations or rural areas of mainland China. The study confirms good primary results in rural areas. Furthermore, the follow-up was 36 months and women who screened positive for HPV received further interventions.

A meta-analysis that included 24 guidelines for the treatment of cervical pre-cancerous lesions and 14 guidelines for the treatment of cervical cancer in China and overseas, covering Asia, Europe, North America, South America, and Oceania, revealed that the guidelines recommend a long-term follow-up plan to monitor disease recurrence after treatment, including HPV testing and/or cytology or colposcopy^27^. Therefore, a cohort study was chosen for follow-up observations. 

In this study, the overall prevalence of hrHPV infection is almost two-fold greater than the 9.91% HPV positive rate detected by Zhao Yanxia et al. with Chinese women in rural areas between 2009 and 2018^21^. These authors also reported 153.88 cervical precancerous lesions/100,000 women and 21.58 cervical cancer/100,000 women, with a 91.24% early cervical cancer diagnosis. Prevalence of HPV 16, HPV 18, and HPV 16/18 are close to that reported by another study^28^, despite the different prevalence of other hrHPV types. Our results show that HPV 16 and HPV 16/18 are the dominant types found in the cytological diagnosis of cervical lesions among women in rural China; therefore, HPV 16 and HPV 16/18 infection have an important role in the prevention and treatment of cervical cancer in rural China.

Consistent with the results of other studies^29^^-^^31^, the cytological diagnosis of patients with hrHPV, HPV 16, HPV 18, or HPV 16/18 genotypes are significantly more severe that those of HPV-negative patients. The difference between our positive hrHPV rates in cytological lesions and those found in the literature^28^ may be due to the low sensitivity of the cytological diagnosis and the lack of determination of the degree of cervical lesions using histopathological results. Because of the geographical environment and sample selection criteria, the infection rates of HPV are different from those with different cytologies^32^. Therefore, the management of patients with abnormal cytological hrHPV infections in cervical cancer screening should be strengthened. 

The frequency of hrHPV-negative women in LSIL is lower to that in the ARTISTIC study (1.14%)^33^; this difference in prevalence may be due to age differences among the participants. The prevalence of hrHPV-negative participants in women with ≥ LSIL is 55.25%; thus, the number of hrHPV−/≥LSIL lesions overlooked by the primary HPV test is extremely low.

The detection rates of CIN2/3+ are higher than the detection rate of pre-cancerous lesions in the two national cancer screening programmes^33^^,^^34^. The detection rates of CIN2/3+ in cervical cancer screening in rural areas may be different from those in other geographical locations due to the used screening tools, characteristics of the parti-cipants, study design, methods (including the classifications used in cytology and pathology), and the principles of biopsy sampling^6^. The prevalence of cervical lesions among the population with a higher screening level is lower than that among the population with lower screening level^35^. 

Consistent with the results of other studies^25^^,^^36^, higher infection rates of hrHPV, HPV 16 and HPV 16/18 associate to higher levels of histological diagnosis among the whole population. Their infection rates in CIN2/3+ and cervical cancer are found to be similar to those in the ATHENA study conducted in the USA^37^ in study areas with high incidence of cervical cancer and certain population characte-ristics such as ethnic minority groups, specific age ranges, and poor healthcare awareness. While HPV 16 is the main dominant type, HPV 18 positivity is low among Chinese women and the infection rate of HPV 18 in CIN2/3+ differs from that reported by Stoler et al.^38^ This may be due to differences in race, population characteristics, and age range, leading to varying HPV 18 infection rates in different cervical lesions.

Some reports indicate that hrHPV-positive women at baseline and follow-up visits have a higher risk of developing CIN2/3+ than with other types of infection at different time points, with the cumulative incidence of CIN2/3+ increasing with longer follow-up times^39^^,^^40^. However, our RRs for CIN2+ for positivity to other 12 hrHPV types are lower than those in other domestic and foreign reports^25^^,^^41^. 

Previous findings^42^ are consistent with CIN2/CIN3+ cumulative incidences in women who screened positive for different genotypes of hrHPV at the follow-up on Month 36, despite being higher than in CIN2+ HPV-positive women in rural China. Any hrHPV positivity increased the risk of CIN2/3+ compared with HPV negativity after the follow-up on Month 36. The CIN2/3+ cumulative incidences for HPV 16 positivity are the highest and for HPV negativity the lowest, which shows the importance of focusing on the management of hrHPV, particularly on the HPV 16 group, for the prevention and treatment of cervical cancer in China. A first cytology positive for HPV 16 increases the immediate risk of a CIN3+ diagnosis, even if the cytology shows NILM; thus, it is sufficient for ordering a colposcopy and indicating the preferred treatment for advanced squamous intraepithelial lesions^43^. However, according to the ASCCP guidelines^43^, colposcopy is also recommended for HPV 18 and HPV 31/33/52/58, considered to have a risk for CIN3+ close to that of HPV 18^43^. As the referral colposcopy threshold was not reached and the cancer risk of HPV 31/33/52/58 is significantly inferior to that of HPV 18, they were uniformly managed by cytological shunts^43^. However, the risk of CIN3+ increases less clearly with HPV 18 infection^44^.

Consistent with previously findings^40^, the cumulative incidence rate of CIN2/3+ for any positive hrHPV is significantly lower compared to that of cytology, which confirms that the sensitivity of HPV screening is higher than cytological screening. 

It is worth noting that CIN2/3+ cumulative incidences for women who screened negative for HPV were 0.5% and 0.2%, respectively, similar to the results obtained in the ATHENA study^37^. Although research shows HPV positivity has an important role in cervical cancer screening, the health of HPV-negative women also requires monitoring.

The present study shows that the cumulative incidence of CIN2/3+ increases alongside the severity of cervical cytology at Month 36 and is lower in lesions ≥ ASCUS negative to HPV than for HPV positivity with ASCUS or NILM, similar to the findings of a previous research^45^ with a longer follow-up (5 and 9 years). Another study, after a 36-month follow-up^46^, reported CIN3+ cumulative incidences for NILM with positivity against HPV, HPV 16, and other hrHPV types of 4.6%, 10.6%, and 2.4%, respectively, which differs from our findings (3.6%, 13.1%, and 9.2%). Therefore, the higher cumulative risk for CIN2/3+ possibly relates to HPV positivity: the cumulative incidence rates of CIN2/3+ in cytological lesions with hrHPV positivity are higher than those in normal cytology with hrHPV positi-vity (particularly HPV 16 and HPV 16/18). Moreover, CIN2/3+ cumulative incidences for NILM with HPV negativity are 0.2% and 0.1%, respectively, that is, lower than those found in the ATHENA study (0.3%)^37^. Hence, the screening interval of cervical cancer in the population with normal cytology and HPV negativity should be appropriately extended to five or more years. Moreover, this may imply that women in rural China have low awareness of health issues and safety.

There are some limitations to this study. First, women who tested positive at baseline did not undergo colposcopies due to the need for additional visits to the clinic, which may have resulted in misdiagnosed cases. Second, following cervical cancer screening guidelines and ethical requirements, patients who screened negative for HPV and ASCUS did not immediately undergo a colposcopy nor required follow-ups, and some patients may have been omitted. Third, we chose rural women who were young and had low literacy levels regarding cervical cancer screening; follow-ups were also a challenge for this population, e.g., some could not attend the scheduled appointments. Another limitation of our study is the high proportion of ASCUS and other cytological abnormalities (above the national average) among the participants. This may be linked to a low quality of the cytology spe-cimens (inaccurate smears), lack of standardised training and quality control for the cytologists, and the high prevalence of other infections or inflammation. Therefore, future studies should improve the quality control of the cytology testing and consider the use of other biomarkers, e.g., p16/Ki-67 dual staining, to improve the specificity and accuracy of the cytology diagnosis.

In conclusion, hrHPV testing, including hrHPV, HPV 16, HPV 18, HPV 16/18, and the other 12 hrHPV types, significantly reduce the risk of CIN2/3+ compared with cytology alone. Therefore, hrHPV testing is an effective primary method for cervical cancer screening that fits public health demands and health resources in China’s rural areas, reducing the risk of the lesions progressing to cancer in these women.
